# One-Step Fabrication Process of Silica–Titania Superhydrophobic UV-Blocking Thin Coatings onto Polymeric Films

**DOI:** 10.3390/biomimetics9120756

**Published:** 2024-12-12

**Authors:** Sharon Hayne, Naftali Kanovsky, Shlomo Margel

**Affiliations:** Department of Chemistry, Institute of Nanotechnology and Advanced Materials, Bar-Ilan University, Ramat-Gan 5290002, Israel; haynesh99@gmail.com (S.H.); nakanovsky90@gmail.com (N.K.)

**Keywords:** polymeric films, surface modification, silica nanoparticles, UV-resistant coatings, thin coatings, mechanical durability, superhydrophobic coatings

## Abstract

Developing a durable multifunctional superhydrophobic coating on polymeric films that can be industrially scalable is a challenge in the field of surface engineering. This article presents a novel method for a scalable technology using a simple single-step fabrication of a superhydrophobic coating on polymeric films that exhibits excellent water-repelling and UV-blocking properties, along with impressive wear resistance and chemical robustness. A mixture of titanium precursors, tetraethylorthosilicate (TEOS), hydrophobic silanes and silica nano/micro-particles is polymerized directly on a corona-treated polymeric film which reacts with the surface via siloxane chemistry. The mixture is then spread on polymeric films using a Mayer rod, which eliminates the need for expensive equipment or multistep processes. The incorporation of silica nanoparticles along with titanium precursor and TEOS results in the formation of a silica–titania network around the silica nanoparticles. This chemically binds them to the activated surface, forming a unique dual-scale surface morphology depending on the size of the silica nanoparticles used in the coating mixture. The coated films were shown to be superhydrophobic with a high water contact angle of over 180° and a rolling angle of 0°. This is due to the combination of dual-scale micro/nano roughness with fluorinated hydrocarbons that lowered the surface free energy. The coatings exhibited excellent chemical and mechanical durability, as well as UV-blocking capabilities. The results show that the coatings remain superhydrophobic even after a sandpaper abrasion test under a pressure of 2.5 kPa for a distance of 30 m.

## 1. Introduction

Artificial systems inspired by nature, or bionics, have been used and adapted for convenience in our everyday lives. Examples of these types of technologies include adhesive materials that can be removed without leaving residue, imitating the feet of the gecko, hook and loop fastening mechanisms, imitating burrs, and imaging applications such as sonar, radar and ultrasound, imitating bat echolocation. Another example of bionics is artificial superhydrophobic surfaces, which are surfaces characterized by their ability to repel water. This adaptation has been inspired by the leaves of the lotus flower and has long been of academic and industrial interest.

Superhydrophobic surfaces, like that of the lotus leaf, are defined by their ability to repel water and are characterized by having a rough surface topography and hydrophobic surface chemical groups. The hydrophobic surface chemistry contains non-polar bonds that lower the surface energy and thus repel water molecules rich in polar hydrogen bonds. Superhydrophobic surfaces have a broad range of applications such as self-cleaning, anti-corrosion and medical devices, making it a focal point of both academic and industrial research [1].

A surface is characterized as superhydrophobic when the water contact angle (WCA), the angle between a tangent of a water droplet and a solid surface, is greater than 150° and the contact angle hysteresis is lower than 10°. Superhydrophobicity results from a delicate interplay between surface roughness and surface free energy. The rough surface topography enhances water repellence by entrapping air pockets within the surface cavities, reducing the interaction between water and the surface [2,3,4]. Many theoretical models describe surface wetting behavior. The Cassie–Baxter model [5] explains air pockets trapped within the rough surface cavities, creating a composite interface of solids and air beneath the water droplet, repelling the water and lowering the WCA. This phenomenon, referred to as the “lotus effect”, allows water droplets to roll off easily while carrying dirt along [6]. By decreasing water interactions and entrapping air, surface roughness is integral to achieving extreme water repellence [7].

Surface free energy is another critical factor affecting surface–water interactions. Surfaces treated with low-surface energy materials, such as fluorinated compounds or long chains of hydrocarbons, reduce the interaction between the water droplets and the surface due to their inherent hydrophobic nature [1]. A surface structure composed of nano and micro scale roughness with a layer of a low-surface energy material is crucial in achieving the desired superhydrophobic characteristics [8,9].

Various materials, techniques and methods have been employed for implementing superhydrophobic properties in a surface. These methods include techniques such as electrochemical deposition [10,11,12], wet chemical reactions [13,14,15,16], electrospinning [17,18,19,20], the dip coating technique [21,22,23,24,25], spray coating [26,27,28,29], layer-by-layer deposition [30,31,32,33,34], lithography [35,36,37,38], chemical vapor deposition (CVD) [39,40,41,42] and nanoparticle self-assembly [43,44]. These approaches create a coating layer with hydrophobic surface chemicals and a roughened topography. However, developing durable, cost-effective superhydrophobic coatings remains a significant challenge due to issues like mechanical wear and environmental exposure.

Recently, silica (SiO_2_) and titanium dioxide (TiO_2_) have emerged as promising materials for superhydrophobic coatings due to their ability to form robust structures. SiO_2_ facilitates nanoscale roughness and bonds with low-energy materials, enhancing water repellence [45,46,47,48,49,50]. Silica is also used as a basis for intrinsically hydrophobic materials that, when cut or abraded, remain hydrophobic without the need for any surface chemical treatment [51,52].

The Stöber process is a well-known method for synthesizing SiO_2_ which typically uses tetraethylorthosilicate (TEOS) as the monomer for the SiO_2_ matrix in acidic or basic aqueous conditions [53]. Under these conditions, TEOS undergoes hydrolysis by water, which forms silanol groups (Si-OH) followed by condensation reactions to form the SiO_2_ network [54].

Titanium dioxide (TiO_2_) is valued for its strong covalent bonding properties, providing resistance to mechanical stress. Titanium precursors such as titanium (IV) butoxide undergo hydrolysis and condensation reactions, like that of SiO_2_, to form a TiO_2_ network [55]. Combining silica and titanium precursors forms Si–O–Ti bonds, which results in a stable composite surface structure where SiO_2_ maintains surface roughness and TiO_2_ strengthens the coating adhesion to the surface, enhancing coating adhesion and durability [56,57].

Despite progress in the fabrication of superhydrophobic surfaces, maintaining coating durability against mechanical stress and environmental exposure remains a challenge [58,59,60]. Wear and abrasion can reduce surface roughness, and low-energy materials may degrade over time.

Industrially, high costs, complexity, and scalability constraints limit the widespread adoption of superhydrophobic coatings. Additionally, weak physical bonds, such as Van der Waals and hydrogen bonds, result in coatings that are easily removed and require frequent reapplication.

To address these challenges, this research uses a modified in situ Stöber process containing tetraethylorthosilicate (TEOS), titanium (IV) butoxide (TBT) and per-fluorodecyltriethoxysilane (FTES). The monomer solution is thinly spread on a corona-treated polypropylene (PP) surface using a Mayer rod. These monomers react with the surface of an oxygenated PP film in acidic aqueous conditions, thus forming a strong and covalently bound matrix between the monomers and the oxygenated surface. This matrix of strong covalent bonds imparts high durability to the coating as opposed to the physical bonds of the coatings described above. Similar research has been performed on this type of modified in situ process to impart other qualities to surfaces, for example, superhydrophobic, antifog and flexible electronics applications [61,62,63,64].

This method offers several advantages: (1) the formation of strong covalent bonds be-tween the surface and coating, which increases the durability of the coating against mechanical stress, (2) no need for expensive equipment prior to or after the coating process, (3) the need for a minimal amount of monomers to be used in the coating, (4) the use of corona treatment to activate the film surface, which negates the need for chemical binding agents, such as primers or adhesives, prior to the coating process, and lastly, (5) the short amount of time needed for the reaction to be completed, which reduces industrial costs. The superhydrophobic surface layer, which has a micro/nano hierarchical structure, functions not only as water repellent, but also displays self-cleaning ability and UV blocking properties, which makes it suitable for various applications. Additionally, this process is versatile, applicable to various polymeric substrates such as polyethylene terephthalate, polyethylene, and polycarbonate. While this study focuses on PP, the method can be extended to other polymeric films.

## 2. Experimental Section

### 2.1. Materials

Non-treated polypropylene (PP) A4 size films were provided by Mapal Plastics Ltd. (Mevo Hamma, Israel). The following materials of analytical grade were purchased from commercial sources: ethanol (EtOH, HPLC), 2-propanol (anhydrous, 99.5%), ammonium hydroxide (NH_4_OH, 28%), tetraethylorthosilicate (TEOS, 99%), titanium (IV) butoxide (TBT, reagent grade, 97%) and 1H,1H,2H,2H-perfluorododecyltriethoxysilane (FDTES, 97%) from Sigma-Aldrich (Rehovot, Israel). Double-distilled water (DDW) was obtained from a Trion TSDI column (Treitel Chemical Engineering, Tel-Aviv, Israel). SiO_2_ nano/micro-particles of various sizes were synthesized in our laboratory by following the well-established Stöber method [65,66]. The as-synthesized SiO_2_ particles were washed in ethanol with three centrifugation ultrasonication cycles to remove impurities and then dried off. A main dispersion solution of SiO_2_ nano/micro-particles was prepared by mixing 0.02%/wt SiO_2_ nanoparticles in isopropanol.

### 2.2. Methods

#### 2.2.1. Preparation of Titanium and SiO_2_-Titanium Dispersions for Superhydrophobic Coatings

SiO_2_-TiO_2_ coating dispersions were prepared by mixing the following in glass vials: 1%/wt SiO_2_ nano/micro-particles of the required size in 0.95 mL isopropanol dispersion, and different amounts of FDTES, TEOS and TBT, as per Table 1. Coating dispersions of TiO_2_ were prepared by mixing isopropanol, FDTES TIIP, as specified in Table 1.

#### 2.2.2. Facile Fabrication of SiO_2_-TiO_2_ Thin Coatings Using a Mayer Rod on Corona-Treated Polypropylene Films

PP films were previously washed with ethanol and dried to eliminate surface contaminants. After cleaning, the films were treated by corona discharge in air, with an applied voltage of 350 V. All PP films used in this research were corona-treated. For each corona-treated PP film, a coating solution was mixed with 100 µL of HCL 1 M and immediately spread using a smooth gauge Mayer rod that was dragged at a controlled speed over the film. To ensure uniformity of the coating, the rod was moved slowly and steadily across the surface, maintaining consistent pressure and speed throughout the process. Excess material was dragged along the surface, leaving behind a thin, even layer. All coatings were dried at 80 °C for 20 min.

### 2.3. Characterization Methods

#### 2.3.1. Coated Polypropylene Films

##### Environmental Scanning Electron Microscope (E-SEM)

The morphology of the coatings on PP films was analyzed using an environmental scanning electron microscope (E-SEM). Energy dispersive X-ray analysis (EDS) attached to the SEM device was also used for semi-quantitative elemental analysis of the TiO_2_/SiO_2_ coatings. Detailed morphological characterization and assessment of coating uniformity were performed with a JSM-840 microscope (JEOL, MA, USA). Prior to examination, the dry-coated polymeric films were attached to a carbon tape on a silicon wafer and sputter-coated with iridium to enhance conductivity.

##### Attenuated Total Reflectance (ATR)

Attenuated total reflectance (ATR) measurements were taken after each coating process on the films. The measurements were performed using a Bruker ALPHA-FTIR QuickSnap (Bruker, MA, USA) sampling module equipped with a platinum ATR diamond module. ATR allows for a direct analysis of surface chemistry by identifying chemical bonds and functional groups on the surface, which is critical for assessing coating uniformity and chemical composition.

##### Atomic Force Microscope (AFM)

Surface topographies and average roughness values of the coated surfaces were investigated by an atomic force microscope (AFM). Measurements were conducted using a Bio Fast Scan scanning probe microscope (Bruker AXS, MA, USA). All images were acquired in Peak Force QNM (quantitative nanomechanical mapping) mode using a Fast Scan C silicon probe (spring constant of 0.45 N/m). Measurements were performed under environmental conditions within an acoustic hood to minimize vibrational noise. Images were captured in the retrace direction with a scan rate of 1.6 Hz and a resolution of 512 samples per line. Nanoscope Analysis software (version 1.7) was used for image processing and thickness analysis, applying the ‘flattening’ and ‘planefit’ functions to each image.

##### X-Ray Photoelectron Spectroscopy (XPS)

X-ray photoelectron spectroscopy (XPS) was used for surface elemental characterization, such as chemical composition, elemental states and coating uniformity. XPS measurements were taken using a Nexsa spectrometer (Derby, England) equipped with a monochromatic, micro-focused, low-power Al Kα X-ray source (photon energy 1486.6 eV). Survey and high-resolution spectra were obtained at pass energies of 200 eV and 50 eV, respectively, with a typical source power of 72 W. The binding energies of all elements were recalibrated by setting the CC/CH component of the C 1s peak to 285 eV.

#### 2.3.2. Surface Wettability

##### Water Contact Angle (WCA) and Rolling Angle (WRA)

Water contact angle (WCA) measurements were conducted using a Goniometer (System OCA, model OCA20, Data Physics Instruments Gmbh, Filderstadt, Germany) at room temperature. Each reported value represents the average of 10 contact angle measurements per sample. A 3 µL droplet of double-deionized water (DDW) was deposited onto the surface using a syringe and was then photographed. The static WCA was determined by analyzing the droplet image with a LaPlace–Young curve fitting tool. For water rolling angle (WRA) measurements, a 30 µL DDW droplet was applied via syringe on the sample in a horizontal position. Reported WRAs are an average of three measurements taken at different locations using the same conditions. The droplets immediately rolled off most samples; therefore, the WRA is presumed to be near zero.

##### Self-Cleaning Ability

To evaluate the self-cleaning ability of the prepared superhydrophobic surfaces, the samples were placed at an inclined angle within a Petri dish, and soil was unevenly spread across their surfaces. While maintaining the incline position, water droplets were gently poured onto the samples. As the soil and dirt were washed away, additional water was poured to further evaluate the superhydrophobic properties of the surfaces.

##### Mechanical and Chemical Durability Tests

Durability and mechanical robustness tests allow for numeric evaluations of the coating stability under physical wear and mechanical stress, which are critical parameters and indicators of the coating’s longevity and outdoor performance. The durability and mechanical robustness of the coatings were assessed via a sandpaper abrasion test [67,68]. A 240-grit silicone-carbide sandpaper served as the abrasive surface. The coated films were positioned with the superhydrophobic side facing the sandpaper, and a normal pressure of 2.5 kPa was applied. The samples were moved back and forth over a 10 cm distance along a ruler in a single direction (20 cm distance equals 1 cycle). The WCA was measured after every cycle of several abrasions until it dropped below 150° for three or more cycles.

The chemical durability of the coatings was evaluated by soaking each sample in aqueous solutions with varying pH levels of 1, 7 and 13, each with and without detergent, for 12 h in a shaker for constat movement.

## 3. Results and Discussion

In the present study, we developed a simple and straightforward method for applying superhydrophobic coatings to polymeric films using a one-step coating process using a Mayer rod. This method is also applicable to most thermoplastic polymers as well as various other substrates such as paper and cardboard, without the need for prior corona treatment. This method allows for extremely rapid sample preparation, as the polymerization occurs simultaneously with the application of the coating on the polymeric surface. As a result, wide films can be coated within seconds. Additionally, this approach is highly cost-effective and economical, as it minimizes material waste, reduces processing time, and eliminates the need for complex equipment.

**Figure 1:**
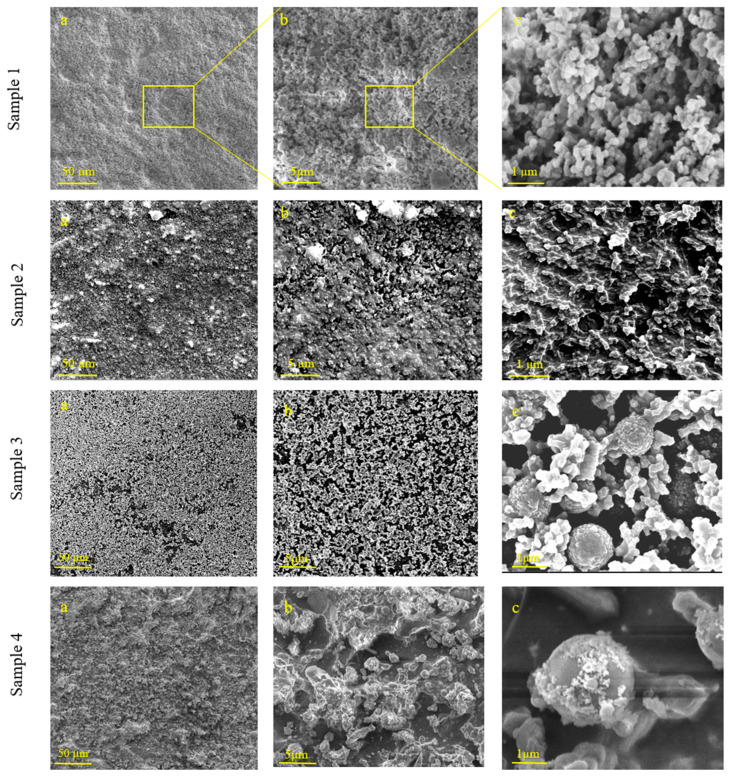
SEM images of coated PP films at several magnifications—coated samples 1–4 with increased magnification of each sample. The scale bars of images (**a**–**c**) are 50 µm, 5 µm and 1 µm, respectively.

As previously discussed, experimental results in the literature demonstrate that a surface exhibits superhydrophobic properties due to a combination between its structural characteristics and its chemical composition. Surface roughness plays a critical role in enhancing hydrophobicity, often leading to superhydrophobicity when combined with low-surface-energy materials. The presence of micro- and nanoscale structures amplifies the water repellence of the surface by reducing the water contact angle due to air trapped between the formed cavities. This effect also contributes to the self-cleaning properties of the superhydrophobic surface. Consequently, surface roughness is a crucial factor in the development of such coatings. We achieved a high surface roughness due to a combination between SiO_2_ nano/micro-particles and silica/titania, which forms smaller-scale structures on top of the SiO_2_ nano/micro-particles. The addition of SiO_2_ particles of various sizes to the coating results in a coating morphology consisting of hierarchical structures (Figure 1), increasing roughness (Table 3), and an increase in the coating durability.

### 3.1. Surface Morphology and Chemical Composition of TiO_2_ and TiO_2_-SiO_2_ Composite Coatings on Polypropylene Films

As previously mentioned, water contact angle is directly affected by the surface roughness and is higher when the surface consists of hierarchical structures. The surface morphology of the various coatings and their microstructures was analyzed by SEM. Figure 1 shows SEM images of samples 1–4, as marked in Table 1. The coating in sample 1 (Figure 1, sample 1, images a–c) was synthesized using titanium isopropoxide and hydrochloric acid, resulting in roughness of the surface. In this sample, the TiO_2_ coating is uniform upon the surface in both coverage and roughness. It seems like the structure of sample 1’s coating is composed of many TiO_2_ clusters with a diameter of about 100 nm stacked one on another, forming a very porous structure that contributed to the water-repellent properties. The highly textured surface plays a pivotal role in affecting the superhydrophobic effect by promoting the Cassie–Baxter model, describing air pockets trapped in the cavities between the surface and the water droplets, resulting in an increase in the water contact angle.

Samples 2–4 contain, in addition to the TiO_2_, also SiO_2_ formation, due to the presence of TEOS. The SiO_2_ nano/microparticles have an average size of 70 nm, 250 nm and 500 nm, respectively. These particles contain a hydrophilic surface, allowing the formed TiO_2_-SiO_2_ network to bond with -OH groups on the SiO_2_ particles’ surface, resulting in covalent bonding between the SiO_2_ particles and the formed TiO_2_-SiO_2_ nucleation centers. The resulting morphology of the TiO_2_-SiO_2_ network on the SiO_2_ particles is called raspberry-like due to the core–shell structure of a large center covered with smaller particles. Due to this hierarchical morphology, the coatings exhibit extremely high WCA and 0° rolling angles (Table 3). The raspberry-like particles are randomly scattered along the surface of the substrate along with the TiO_2_-SiO_2_ amorphous structures between them, covering the entire area.

**Figure 2:**
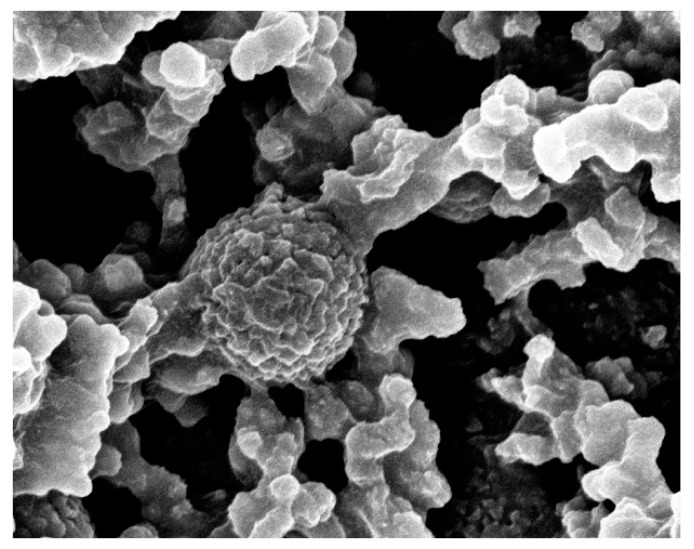
SEM image high magnification of sample 3: hierarchically structured surface composed of composite of 250 nm SiO_2_ particles with titania–silica structures, forming raspberry-like particles.

To illustrate the raspberry-like morphology and the development of the hierarchical structure, a higher-magnification image of sample 3 is presented in Figure 2. The SEM micrographs in Figure 1 and Figure 2 distinctly reveal the hierarchical coating topography on the films’ surface. These images indicate that the raspberry-like particles create a topography with micro- and nanoscale features capable of entrapping air, thereby producing superhydrophobic surfaces characterized by exceptionally high water contact angles (WCAs) and low water roll-off angles (WRAs).

**Figure 3:**
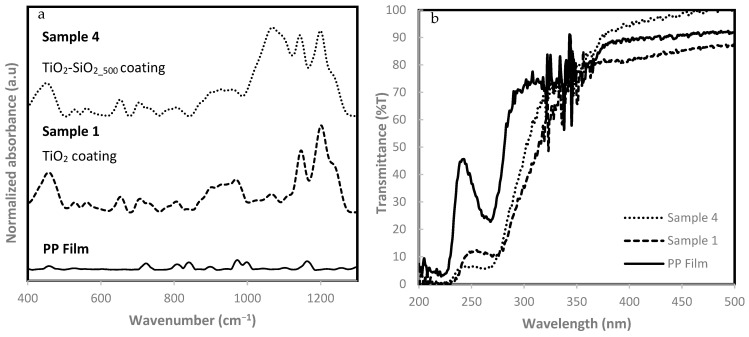
FTIR absorbance spectra (**a**) and UV transmission spectra (**b**) for PP films, samples 1 (TiO_2_ coating) and 4 (TiO_2_-SiO_2_500_ coating).

**Figure 4:**
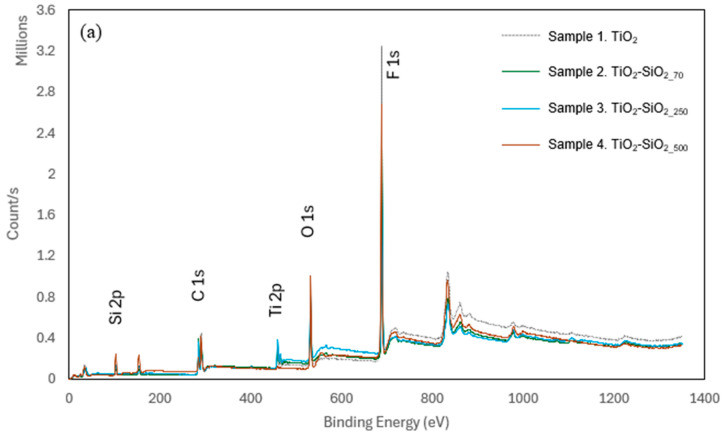

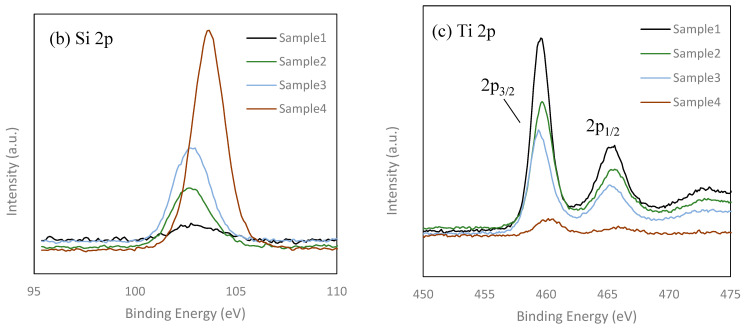
XPS spectra of samples 1–4 (**a**), HR-XPS spectra of samples 1–4 of Si 2p (**b**) and of Ti 2p (**c**).

The presence of titania and Ti-Si functional groups on the surface can be confirmed by FTIR/ATR analysis (Figure 3) and XPS analysis (Figure 4, Table 2). Figure 3a shows the FTIR spectra of a corona-treated uncoated PP film, the sample 1 coating of TiO_2_ on PP and the sample 4 coating of both silicon and titanium on PP. In the baseline spectrum of an uncoated PP, which serves as a reference, there is minimal absorption below 1000 cm^−1^ and the characteristic C-H bending and rocking vibrations of the polymer backbone are expressed as weak peaks in the region between 700 and 900 cm^−1^. The FTIR spectra of samples 1 and 4 contain absorption bands in the lower region of the spectra around 450–750 cm^−1^, which is attributed to the presence of titanium in the coatings. Specifically, the peaks around this area at 480, 670 and 735 cm^−1^ are attributed to Ti-O bond vibrations, symmetric O-Ti-O stretch, and non-symmetric vibrations of Ti-O-Ti during tetrahedral coordination, respectively [69], as the broad peak at 1042 cm^−1^ is usually referred to as the Si-O-Ti bridge vibrations [70]. The spectra of sample 4 contain very significant absorption bands between 1000 and 1300 cm^−1^, where both Si-O-Si and Si-OH stretching vibrations occur. The absorbance peak observed at 1080 cm^−1^, specifically in the spectrum of sample 4, may correspond to the Si-O-Si stretching vibrations, indicating the presence of silane crosslinking, which barely exist in both other graphs due to the lack of silica particles and TEOS. Those peaks can be attributed to Si-O-Si bonding forming a vast SiO_2_ network during polymerization directly on the PP surface. When comparing the spectra of samples 1 and 4, it seems that the incorporation of SiO_2_ in the coating leads to a more structured and interconnected coating, based on the increased intensity of the peaks in this region and their complexity. The FDTS presence is also indicated by the C-F stretching vibrations’ significant peaks at 1810 and 1210 cm^−1^, corresponding to the C-F stretching vibration and bending band in the fluorinated alkyl chains, respectively. The peak at 1230 cm^−1^, appearing as a shoulder to one of the C-F peaks, can be ascribed to the stretching vibrations of the Si-OH bonds, which also appears on sample 1’s spectra due to the presence of silicon atoms originating from FDTS [71]. The coated films show characteristic peaks related to the functional groups of the titanium, fluorosilane, and SiO_2_ compounds, confirming the successful deposition of the superhydrophobic coatings. The presence of C-F and Si-O-Si bonds, alongside the Ti-O bonds, highlights the formation of a complex, functionalized surface on the polymeric substrate.

Assessment of the coatings’ effectiveness in blocking UV radiation was carried out by transmittance measurements. The UV transmission spectrum of samples 1, 4 and plain PP is described in Figure 3b. Since all coatings are translucent and have no color, the transmittance in the visible wavelength area between 400 and 700 nm is around 100%. The transmittance spectrum of the uncoated PP film indicates a higher transmittance in relation to the coated samples, especially around 250 nm, reaching almost 50% transmittance. It is well established that PP has a limited ability to block UV radiation, especially in the UVA region (320–400 nm) [72]. In contrast to the PP film, both coated samples demonstrate significant UV absorption, not higher than 10% in the region below 280 nm. The contribution of the coating to the UV radiation absorbance is visible right below 350 nm, where the PP absorption remains around 80%, while the coatings show a steep increase in absorbance. The enhanced UV-blocking capability of this coating can be attributed to the presence of titanium, which is known for its strong absorption of UV light, particularly in the UVB and UVC regions. Si also plays a role by contributing to the film’s overall stability and durability without significantly affecting the transmittance in the visible range. The presence of silica and titania in one coating combines the UV blocking properties of the titania as well as the increased durability-resistant properties of silica. Sample 1 exhibits better UV absorption, especially in the UVC region (below 280 nm) where the transmittance is about 6%. For all samples, the transmittance is lower only in the region of the UV section. However, the transmittance increases when reaching the visible radiation area. This demonstrates that, in addition to the excellent ability of the coatings to block UV radiation, they do not harm the transparency and clarity of the PP film in the visible spectrum.

XPS is a widely used technique for quantitative surface analysis. Here, XPS was carried out to determine the surface composition of each coated PP film and to assess the influence of the amount of titanium in relation to the silica particles on the coating structure, as seen in Figure 4a. Across all spectra, peaks representing silicon, titanium, oxygen and silica were prominent and consistent with the coating materials used (Table 2). Carbon signals were observed in all samples and in similar percentages. The typical X-ray penetration depth is up to 5 nm, meaning that the carbon presence in the samples originates mostly from the PP substrate and trace amounts from the fluorinated silanes present in the coating. The titanium signal corresponding to the Ti2p binding energies confirms the presence of titanium dioxide (Ti^4+^); the Ti2p_3/2_ peak at 459.8 eV of Ti(IV) in TiO_2_ can be seen in all samples in Figure 4c. Comparing Figure 4a,b allows us to consider that the percentage of Ti2p is higher as the silica content is lower, meaning that the signal corresponds to a greater surface coverage of titania with lower presence of the SiO_2_ nanoparticles. This also corresponds with the SEM images showing a partial surface coverage of the titania with increased size of silica particles. The signal for silicon is strongest in sample 4, which contains the largest silica nanoparticles (500 nm), as opposed to the smaller 70 nm SiO_2_ nanoparticles used in sample 2. Sample 1, containing TiO_2_ without SiO_2_ particles, exhibits traces of Si2p, which corresponds to the Si atoms of FDTS. These findings align with the expected chemical compositions and underscore the flexibility of the coating methods in tailoring surface properties for diverse applications.

### 3.2. Wettability Properties

**Figure 5:**
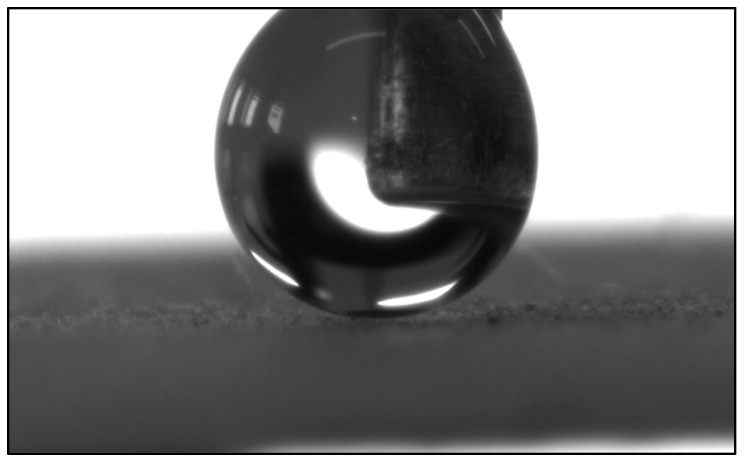
Water contact angle measurements for samples 1–4. The droplet is being forced onto the surface using the needle, which yields a perfectly spherical droplet with 180° WCA.

The wettability properties of the coatings were studied by sessile water contact angle measurements of the films. Surface roughness plays a crucial role in affecting the water contact angle and superhydrophobicity. This feature can be controlled by changing the size of silica nanoparticles used in each coating. Incorporating silica nanoparticles allows varying degrees of surface roughness depending on the size of the particles. The size of the SiO_2_ nano/micro-particles combined with TiO_2_ or with the TiO_2_-SiO_2_ network formed on the surface was designed to form a dual-scale roughness to achieve superhydrophobicity. Table 3 shows the AFM measurements of the topography and average surface roughness values of each coated film alongside the water contact angle and rolling angle. Incorporation of larger-sized SiO_2_ particles increases the surface roughness. Despite some differences in surface roughness, all samples showed excellent superhydrophobicity, as the water droplets immediately rolled off the horizontally placed surface (Figure 5). Sample 1, containing only TiO_2_, had the lowest surface roughness of 5.4 nm as opposed to the larger surface roughness that was observed for sample 4. This is due to the addition of SiO_2_ nano/micro-particles to the coating in which smaller nanoparticles (70 nm) were added to sample 1 and larger particles (500 nm) were added to sample 4. According to the SEM images presented in Figure 1, the morphologies of the coatings are dependent on the size of the nano/micro-particles added to the coating solution. The addition of larger SiO_2_ particles to the coating solution presents as raspberry-like structures, while the addition of smaller particles presents as tunnel-like structures on the surface. These structures increase the surface roughness of the coating and amount of air that can be trapped within the cavities, which results in extremely high water contact angles and very low rolling angles.

### 3.3. Self-Cleaning Ability

**Figure 6:**
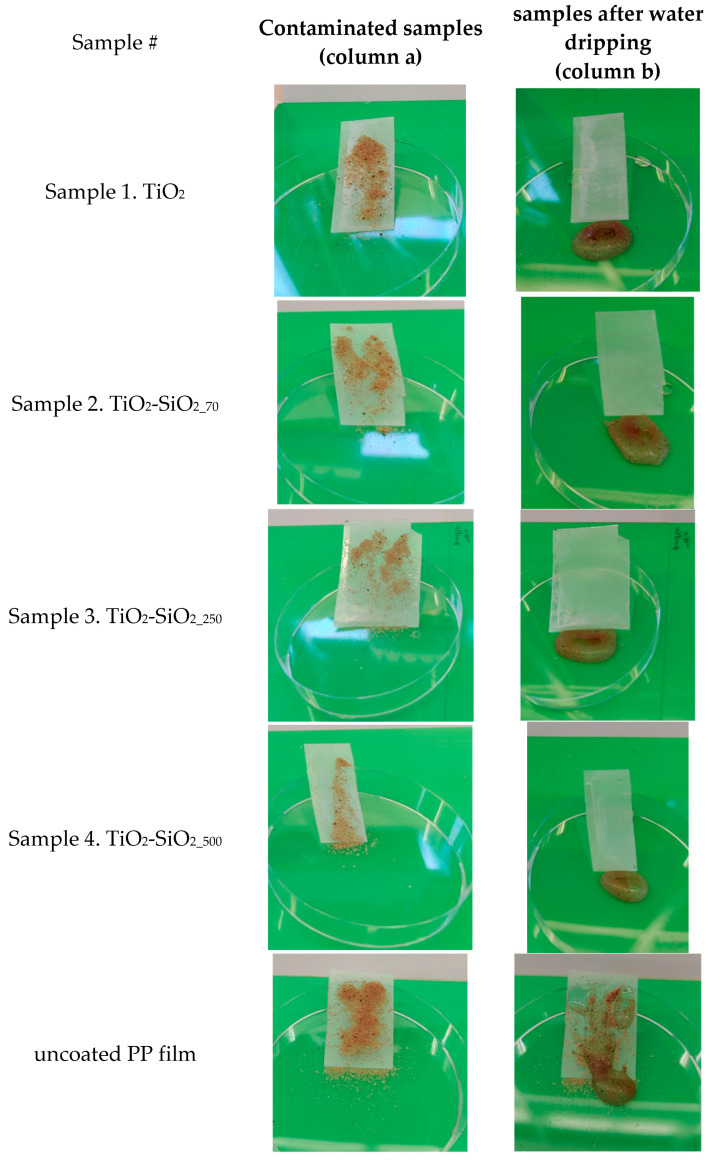
Samples 1–4 contaminated with soil (**column a**) and after self-cleaning test (**column b**).

The self-cleaning performance of superhydrophobic samples 1–4 was evaluated using sand as a model contaminant. Figure 6 illustrates the self-cleaning process, where sand powder was unevenly distributed over the coated samples and plain PP film, followed by the application of water droplets onto the contaminated surfaces. On the coated samples, the rolling water droplets effectively captured and removed the sand particles, leaving the surfaces clean. In contrast, on the plain, uncoated PP film, the sand particles were displaced but remained mixed with residual water on the surface, resulting in a dirtier appearance.

### 3.4. Mechanical Properties

There is no single standardized test for durability properties of superhydrophobic coatings. The absence of a universally accepted method to test such coatings makes the comparison of results across various methods very difficult. Despite significant advances in the development of superhydrophobic coatings, one major gap is their lack of wear resistance and durability. Maintaining water repelling properties under physical stress remains a challenge that is mostly overcome by multi-step processes that involve long, complex, and expensive methods such as multi-step electrodeposition [73], LbL (layer-by-layer) assembly of nanoparticles and polymers [74] and many more [75,76,77]. These methods offer improvement in superhydrophobic performance but are difficult to perform and control in large-scale applications. Here, we report on a single-step method that requires no special or expensive equipment and yields excellent superhydrophobic properties.

**Figure 7:**
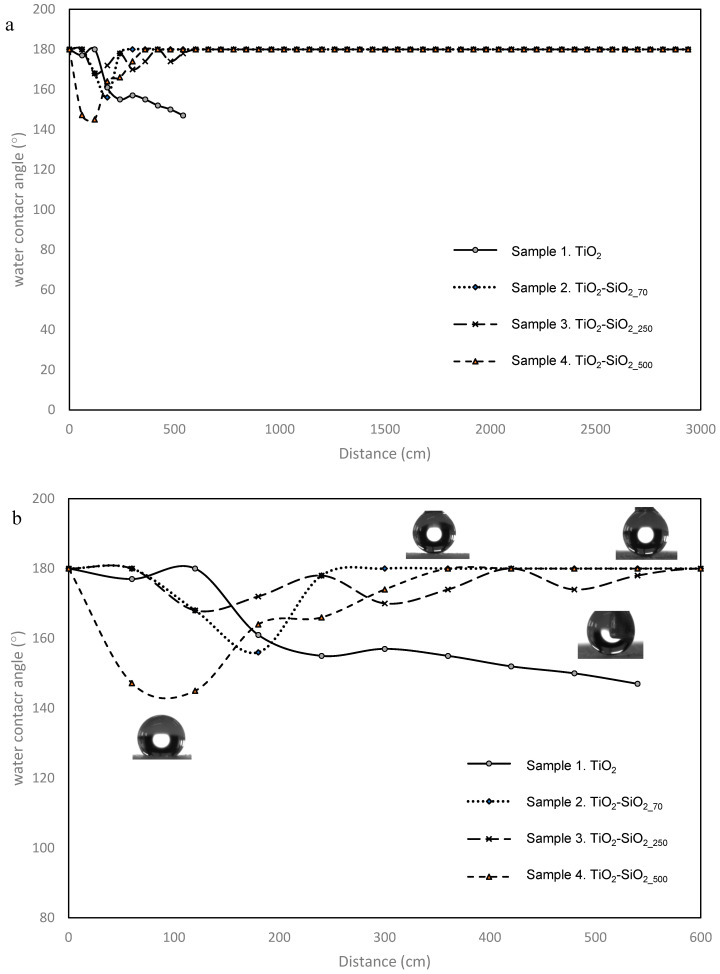
Water contact angles of coatings 1–4 after (**a**) 3000 m of the sandpaper abrasion test and (**b**) zoom in on the WCA results up to 600 m.

The issue of the coating’s durability was addressed by first performing surface preparation on the PP films prior to the coating and then by reacting TIIP, TEOS and SiO_2_ nano/micro-particles to the surface, allowing for covalent bonding between the PP surface and the coating. As mentioned in the experimental section, the durability of each coating on PP films was assessed using the sandpaper abrasion test. A weight was placed on the back of a coated film facing down on a 240-grit silicone-carbide sandpaper. This weight applied a pressure of 2.5 kPa between the coating and the sandpaper. Durability resistance results of each coating can be seen in Figure 7. The graphs in Figure 7 presents the WCA measurement after each rubbing cycle applied to each coating. Sample 1, containing only TiO_2_, shows the lowest durability resistance in relation to the other samples containing both TEOS and SiO_2_ nano/micro-particles. Although this sample exhibits a relatively constant CA, after 60 cm, there is a gradual degradation and the sample loses superhydrophobicity after 300 cm. Sample 3, containing 250 nm SiO_2_ particles, experienced a dip at the beginning of the test, but showed an increase in in WCA back to 180°. Samples 2 and 4 contain SiO_2_ nano/micro-particles with larger sizes of 70 and 500 nm, respectively. Both show very impressive durability resistance and maintain their superhydrophobic properties. The test was performed with samples 2–4 up to a distance of 30 m without any WCA reduction.

The combination of corona–discharge pretreatment on the PP films prior to the coating application along with incorporation of TEOS and nano/micro-particles seems to improve the adhesion of the coating through silane chemistry. The reactivity of the PP increases due to the introduction of polar groups on the surface [50]. The reactive silanol groups that form on the SiO_2_ particle surface and the hydrolyzed form of TEOS can react with the functional groups on the corona-treated surface. This forms strong covalent bonds and results in enhancing the overall adhesion of the coating [67]. The presence of the SiO_2_ particles increases the coating’s durability to mechanical scratching effectively. TEOS also facilitates the formation of a crosslinked SiO_2_ and a silica–titania network within the coating, which provides improved resistance to chemical and physical degradation [78,79]. TEOS acts as a binder that integrates all the components (the SiO_2_ particles and the titanium precursor) into a cohesive network, improving both the superhydrophobicity and the durability of the coatings [80,81]. The decrease in the hydrophobicity of the coatings at the first stages can be related to the partial removal of the coating’s top layers in a nonuniform way, as the sandpaper scratches the surface, forming certain patterns of stripes and grids. Removal of the top coating layer decreases the content of fluorocarbons on the surface, thus resulting in a less hydrophobic surface, which is expressed as an initial lowering of the contact angle. However, Figure 7 shows an increase in the contact angle after applying more sandpaper tests to the coated substrate. This can be attributed to the formation of a new surface topography of the grid-formed structure and the nano-microscale of the coating. At some point, the sandpaper reveals SiO_2_ particles that can slow down the mechanical wear; thus, the new morphology remains stable even after many applications of the sandpaper test. This phenomenon highlights the importance of hierarchical surface designs in durable superhydrophobic coatings, where both surface chemistry and multi-scale roughness contribute to long-lasting water repellency even under mechanical stress. The roughness supplied by the SiO_2_ particles combined with the silica–titania network provides mechanical stability and resilience while maintaining the hierarchical roughness and morphology of the coating, allowing the superhydrophobic properties to be stable. The formation of covalent bonds between the TEOS-derived silica network and the functional groups introduced by corona discharge, combined with the mechanical reinforcement provided by SiO_2_ particles, significantly improves the coating’s adhesion, structural integrity and resistance to wear.

**Figure 8:**
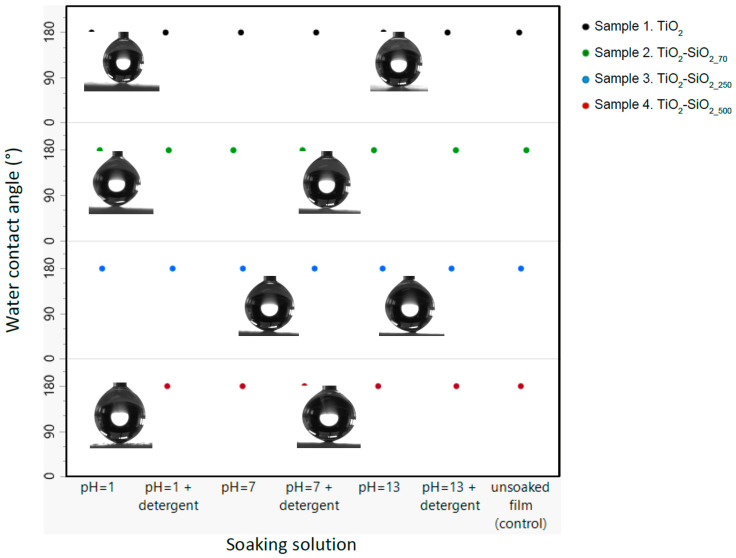
Water contact angle of samples 1–4 before (control) and after soaking in various solutions.

In addition to the mechanical durability testing, the coating’s chemical stability and durability was tested by soaking each type of coated film in solutions with different pH levels, each with and without detergent, for 12 h. The water contact angles of the coatings were tested before and after the soaking, as can be seen in Figure 8. All samples showed no discernible change after 12 h of soaking and no change in coating uniformity in all pH values with or without detergent. This multifunctional approach offers a promising pathway for creating durable superhydrophobic coatings with potential applications in various industries.

## 4. Summary and Conclusions

The present study presents a novel approach for fabricating superhydrophobic coatings on polymeric films that exhibit UV-blocking and wear-resistant properties. The challenge of superhydrophobic coatings that are multifunctional as well as durable is addressed, using a scalable and cost-effective single-step method. Different surface morphologies were achieved in relation to the size of the SiO_2_ particles incorporated. Coatings containing only TiO_2_ or small-sized SiO_2_ nanoparticles seem to organize as tunnels with nanogaps between them, thus increasing the surface area and roughness of the coating. The surface morphology of coatings containing larger-sized SiO_2_ nano/micro-particles appears to contain tunnels composed of silica–titania network structures alongside raspberry-like particles consisting of a SiO_2_ particle core and a silica–titania rough shell. Both forms of coating morphologies and structures show significantly high water contact angles, as the droplets did not stay on the surface but immediately rolled away, exhibiting a rolling angle of 0° and a contact angle of 180°.

Several coatings were made with different compositions to examine how SiO_2_ particles of various sizes affect the surface morphology, roughness and functionality of the resulting coating. It was shown that the incorporation of nano/micro-particles of various sizes affects surface roughness and increases the coating durability from both environmental and chemical wear. These coatings retained their superhydrophobic properties even after being rubbed against sandpaper for a significant distance of 30 m under a pressure of 2.5 kPa. The addition of a titanium precursor to the coating’s solutions imparts UV blocking functionality, making the coating suitable for outdoor use. The combination of mechanical durability, UV resistance, and ease of application makes this coating system highly suitable for a wide range of industrial and commercial applications.

## Figures and Tables

**Table 1:** Volumes of reagents used for each coating solution.

Sample #	SiO_2_ Nanoparticle Average Size (nm)	SiO_2_/Isopropanol Dispersion (mL)	Isopropanol (mL)	FDTES (µL)	TEOS (µL)	TBT (µL)
1. TiO_2_	None		0.91	36.1		45.9
2. TiO_2_-SiO_2_70_	70	0.95		23	7	14
3. TiO_2_-SiO_2_250_	250	0.95		23	7	14
4. TiO_2_-SiO_2_500_	500	0.95		23	7	14

**Table 2:** XPS analysis of atomic percentages of chemical elements in each coating.

Sample #	Si2p	Ti2p	F1s	O1s	C1s	N1s
1. TiO_2_	0.22	4.68	52.38	9.58	32.49	0.55
2. TiO_2_-SiO_2_70_	3.61	3.89	40.7	13.65	37.41	0.74
3. TiO_2_-SiO_2_250_	5.2	2.99	36.51	21	33.18	1.12
4. TiO_2_-SiO_2_500_	9.84	0.4	44.38	20.43	24.51	0.43

**Table 3:** Average roughness (Ra) and static water contact angle (CA) values for plain PP and for coatings 1–4 on PP as a function of silica nanoparticle size in the coating.

Sample #	R_a_ (nm)	CA (°)
Corona-treated PP film	8.7 ± 0.6	54.3 ± 1.2
1. TiO_2_	5.4 ± 2.2	>180
2. TiO_2_-SiO_2_70_	8.9 ± 0.4	>180
3. TiO_2_-SiO_2_250_	22.6 ± 6.3	>180
4. TiO_2_-SiO_2_500_	36.4 ± 5.6	>180

## Data Availability

The original contributions presented in the study are included in the article, further inquiries can be directed to the corresponding authors.
